# Fish Waste—A Novel Bio-Fertilizer for Stevia (*Stevia rebaudiana* Bertoni) under Salinity-Induced Stress

**DOI:** 10.3390/plants13141909

**Published:** 2024-07-11

**Authors:** Zahra Mahdavi, Behrouz Esmailpour, Rasul Azarmi, Sima Panahirad, Georgia Ntatsi, Gholamreza Gohari, Vasileios Fotopoulos

**Affiliations:** 1 Department of Horticulture, Faculty of Agriculture and Natural Resources, Mohaghegh Ardabili University, Ardabil 5619911367, Iran; memahdavi2016@gmail.com (Z.M.); r_azarmi@uma.ac.ir (R.A.); 2 Department of Horticultural Sciences and Landscape Engineering, Faculty of Agriculture, University of Tabriz, Tabriz 5166616471, Iran; s.panahirad@tabrizu.ac.ir; 3 Laboratory of Vegetable Production, Department of Crop Science, Agricultural University of Athens, Iera Odos 75, 11855 Athens, Greece; ntatsi@aua.gr; 4 Department of Horticultural Sciences, Faculty of Agriculture, University of Maragheh, Maragheh 551877684, Iran; gohari.gh@maragheh.ac.ir; 5 Department of Agricultural Sciences, Biotechnology and Food Science, Cyprus University of Technology, Limassol 3036, Cyprus

**Keywords:** abiotic stress, physiological attributes, stevia, fish waste, bio-fertilizer

## Abstract

Currently, different strategies, including the application of bio-fertilizers, are used to ameliorate the adverse effects posed by salinity stress as the major global problem in plants. Fish waste is suggested as a novel bio-fertilizer to mitigate the effects of biotic and abiotic stresses. In this investigation, an experiment was conducted to investigate the effects by applying different concentrations (0, 5, 10, and 15% (*v*/*v*)) of fish waste bio-fertilizer on stevia plants grown under salt stress conditions (0, 20, 40, and 60 mM of NaCl). Results showed that salinity negatively affected growth parameters, the photosynthetic pigments, the relative water content, and the chlorophyll fluorescence parameters while increased the activity of antioxidant enzymes, total phenol, hydrogen peroxide (H_2_O_2_), malondialdehyde (MDA), proline, and total carbohydrates compared with control samples. On the other hand, the application of fish waste bio-fertilizer mitigated the effects of salinity stress by enhancing growth and mitigating stress-relative markers, especially at the highest salinity level (60 mM). Overall, fish waste bio-fertilizer could be considered a sustainable, innovative approach for the alleviation of salinity stress effects in plants and, in addition, fish waste bio-fertilizer did not cause more salinity issues, at least with the applied doses and experiment time, which is an imperative aspect.

## 1. Introduction

*Stevia rebaudiana* Bertoni (stevia) is a perennial medicinal plant belonging to the Asteraceae family. Stevia plants contain many compounds, importantly steviol glycosides (e.g., stevioside and ribosediosides A), in the leaves that are used as natural sweeteners in the food industry [1]. These natural sweeteners are 30 to 400 times sweeter than sucrose and calorie-free. More importantly, since the human body cannot digest these compounds, they are used to prevent diabetes, high blood pressure, fungal diseases, etc. Furthermore, this plant is rich in minerals, proteins, fibers, and essential oils (e.g., caryophyllene oxide and spathulenol as main constituents) [2,3].

Salinity stress, as one of the main abiotic stressors in the world, affects the crop production of most regions in all climate zones [4]. Soluble salts in saline soils are composed of several ions (Na^+^, Ca^2+^, Mg^2+^, Cl^−^, SO_4_^2−^, and HCO_3_^−^), resulting in destructive effects in plants [5]. The adverse effects of salinity on plant growth and performance can be related to the induced osmotic stress [6] and ionic imbalance related to the high presence of sodium (Na^+^) and chloride (Cl^−^) ions, leading to the reduced uptake of potassium (K^+^), calcium (Ca^2+^), magnesium (Mg^2+^) and nitrate (NO_3_^−^) ions [7]. Furthermore, salinity leads to an excessive production of reactive oxygen species (ROS), thereby causing oxidative stress [8,9,10]. Salinity stress causes an increase in biochemical compounds, antioxidant activity, anti-inflammatory activity, and antimicrobial activity [11]. In addition, salinity has negative impacts on seed germination, photosynthesis, transpiration, chlorophyll, carotenoids, chloroplasts, PSII photosystems, and stomatal conductance [5]. Salinity decreased growth, photosynthetic pigments, and relative water content (RWC) and increased proline, total phenolic content, antioxidant activity, antioxidant enzymatic activities, H_2_O_2_, MDA, electrolyte leakage (EL), and essential oil content as well as stevioside and rebaudioside A constituents of essential oils in stevia [1]. Plants tolerate salinity by accumulating osmolytes (e.g., proline or sugar), regulating ion homeostasis, and increasing antioxidant system activity. Nevertheless, the responses and defense strategies of plants to survive and also maintain growth are extremely complex and involve multiple pathways [12].

Organic fertilizers (bio-fertilizers) reduce chemical fertilizer input and improve the chemical structure and biological activity of soils, leading to increased crop yield by assisting in nutrient uptake and ionic balance, particularly under stressful conditions [13]. Fish waste production is of global concern [14]. Fish waste has a high content of proteins, amino acids, peptides, collagen, minerals, enzymes, and other valuable compounds [15]. Therefore, it can be used as a bio-fertilizer. Fish waste as a bio-fertilizer stimulates plant growth by providing amino acids and a slow release of essential macro- and micro-nutrients, and preventing nutrient leaching [16,17]. Amino acids are key elements needed for plant growth and initiate a number of cellular processes, such as the production of indole acetic acid [18]. Fish waste bio-fertilizer contains N-P-K in a 10-6-2 ratio [19]. Fish waste bio-fertilizer increased the growth of eggplants [16]. Bio-fertilizer foliar application supplied P and K^+^ required for plant growth with a poor rooting system under stress conditions [20].

Fish waste production is increasing globally, leading to the disposal of a high content of nutrients and amino acids, and so the collection of the waste and production of liquid bio-fertilizer have become an ambitious project for the fish industry. Accordingly, the purpose of this study was to evaluate the effect of the foliar spraying of liquid fish waste bio-fertilizer, as a food supplement, on key morphophysiological and biochemical characteristics of stevia plants grown under non-stress and salinity stress conditions. To the best of our knowledge, inadequate information is available about the effect of fish waste bio-fertilizer on salinity conditions, likely marking the current study as a step forward toward its effect on plants under stress condition.

## 2. Results

### 2.1. Morphological Traits

**Figure 1:**
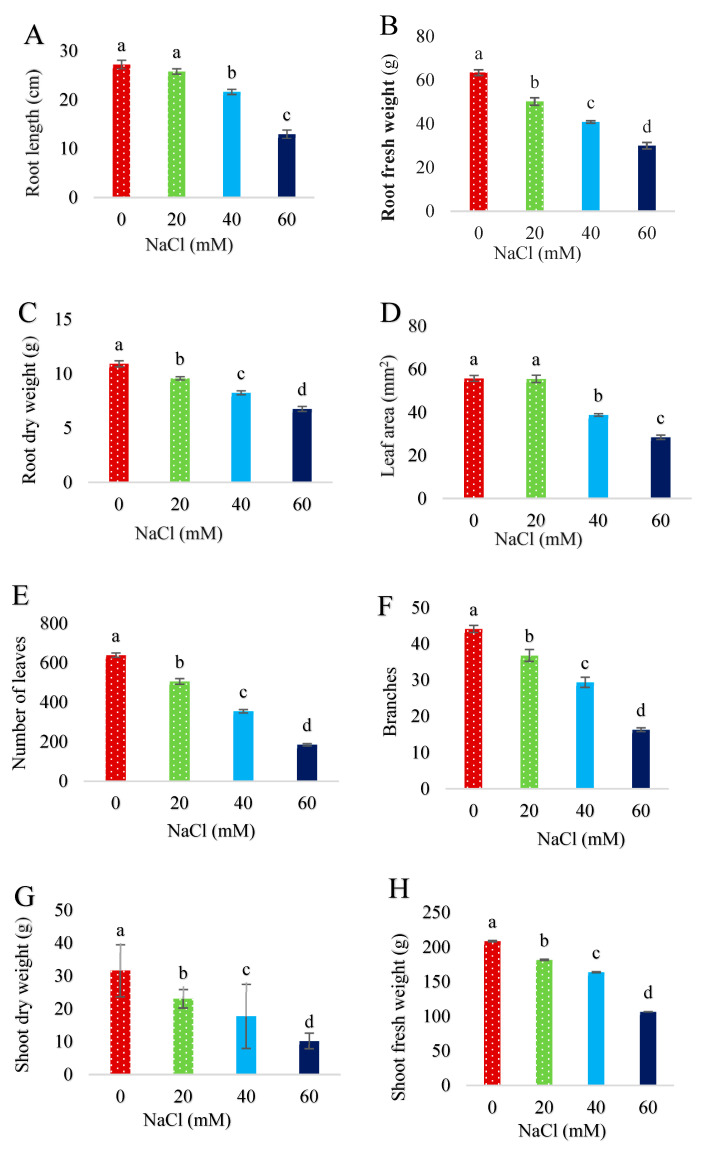
Effect of various concentrations of NaCl on (**A**) root length, (**B**) root fresh weight, (**C**) root dry weight, (**D**) leaf area, (**E**) number of leaves, (**F**) branches, (**G**) shoot dry weight, and (**H**) shoot fresh weight of *Stevia rebaudiana* Bertoni. Same letters indicate no significant differences (*p* < 0.05) based on Duncan’s Multiple Range test.

**Figure 2:**
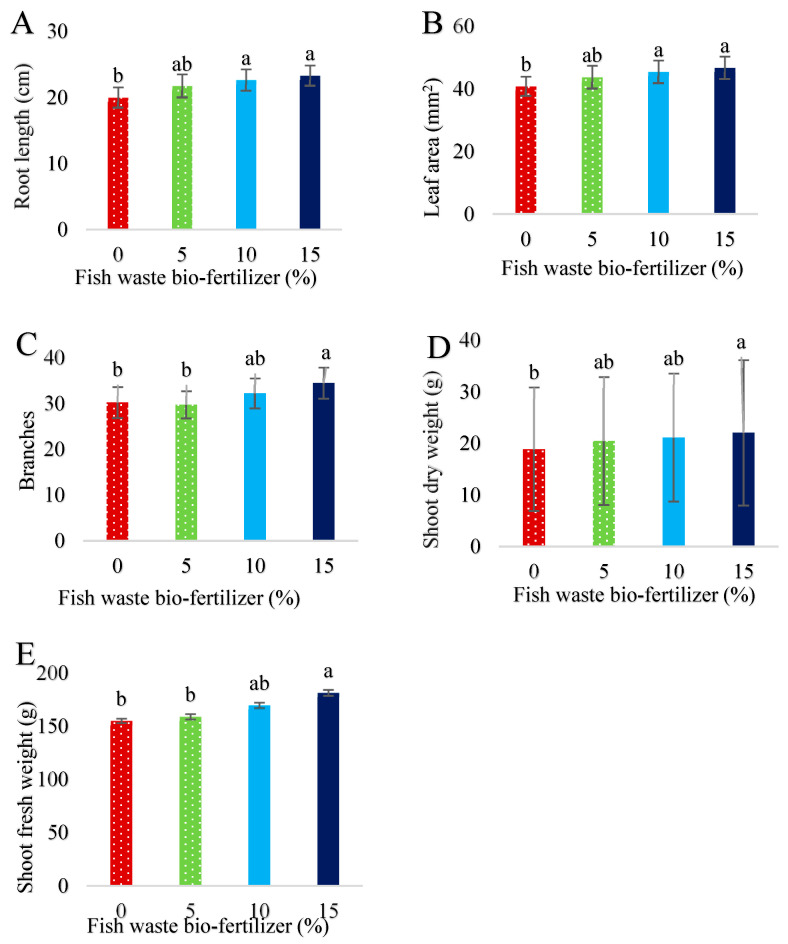
Effect of various concentrations of fish waste bio-fertilizer on (**A**) root length, (**B**) leaf area, (**C**) branches, (**D**) shoot dry weight, and (**E**) shoot fresh weight of *Stevia rebaudiana* Bertoni. Same letters indicate no significant differences (*p* < 0.05) based on Duncan’s Multiple Range test.

**Figure 3:**
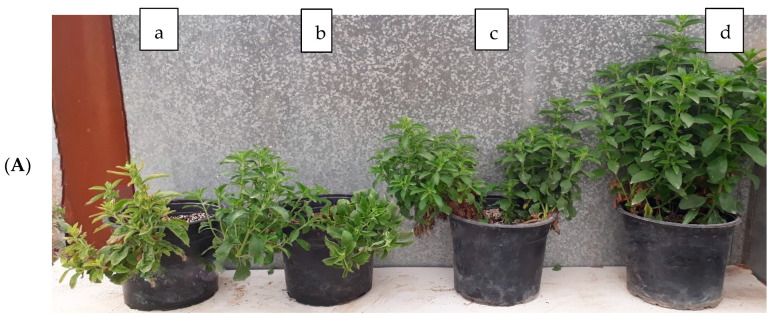

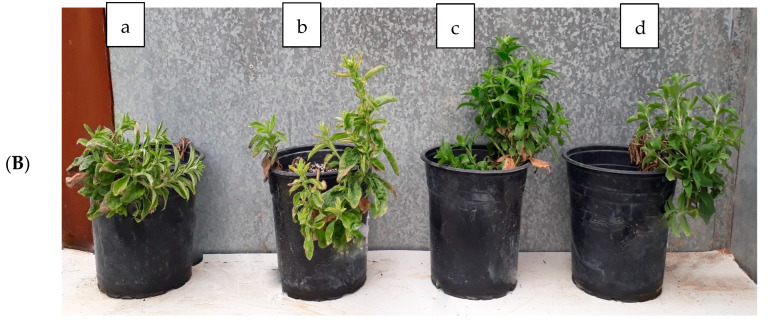
(**A**) Effect of various concentrations of NaCl stress (a: 60 mM, b: 40 mM, c: 20 mM, and d: Control) on *Stevia rebaudiana* Bertoni under no fish waste bio-fertilizer treatment. (**B**) Effect of various concentrations of fish waste bio-fertilizer treatment (a: control, b: 5% (*v*/*v*), c: 10% (*v*/*v*), and d: 15% (*v*/*v*)) on *Stevia rebaudiana* Bertoni under 60 mM NaCl.

Salinity stress significantly (*p* < 0.05) decreased plant growth traits (Figure 1A–F). Fish waste bio-fertilizer significantly (*p* < 0.05) increased root length (Figure 2A) and leaf area (Figure 2B) at 10 and 15% concentrations, and branches (Figure 2C) and fresh and dry weight of shoots (Figure 2D,E) at a 15% concentration. According to the results, fish waste bio-fertilizer at a concentration of 15% achieved optimal results, leading to an increase of 14.54% in fresh weight of shoots, 14.44% in dry weight of shoots, 12.64% in leaf area, and 14.27% in root length compared with control samples (Table S1). Figure 3 presents salinity effects on plant morphological traits (Figure 3A) as well as fish waste bio-fertilizer on the traits under 60 mM salinity (Figure 3B).

### 2.2. Photosynthetic Pigments and ^Fv^/_Fm_

**Figure 4:**
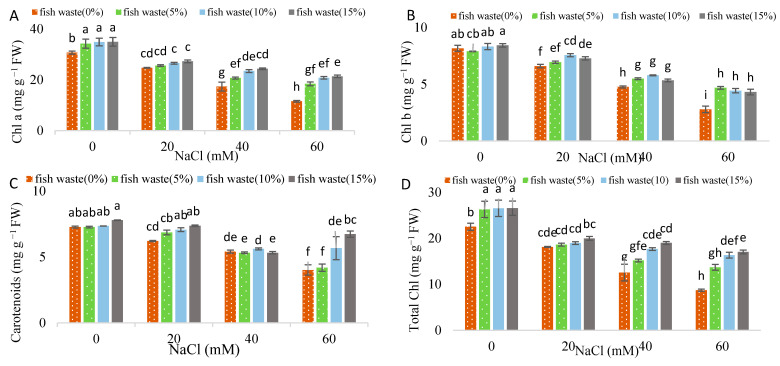

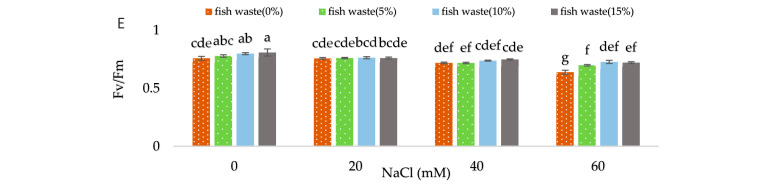
Effect of various concentrations of fish waste bio-fertilizer on photosynthetic parameters ((**A**) Chl a, (**B**) Chl b, (**C**) carotenoids, (**D**) total Chl, and (**E**) ^Fv/^_Fm_) of *Stevia rebaudiana* Bertoni under salinity stress conditions (0, 20, 40, and 60 mM NaCl). Same letters indicate no significant differences (*p* < 0.05) based on Duncan’s Multiple Range test.

Salinity at a 60 mM concentration caused a decrease of 61.27% in Chl a (Figure 4A), 65.85% in Chl b (Figure 4B), 62.46% in total Chl (Figure 4D), 44.84% in carotenoid (Figure 4C), and 15.78% in ^Fv/^_Fm_ (Figure 4E) in comparison with control. Fish waste bio-fertilizer, at all applied concentrations, enhanced total Chl and Chl a, while ^Fv^/_Fm_ increased using 10 and 15% concentrations under non-stress condition. Under 20 mM NaCl, higher Chl b and carotenoid content was recorded using 10 and 15% bio-fertilizer. Under 40 mM NaCl, all concentrations of fish waste bio-fertilizer increased total Chl and Chl b, while 10 and 15% concentrations enhanced Chl a content. Under 60 mM NaCl, all concentrations of bio-fertilizer enhanced Chl a, Chl b, and ^Fv^/_Fm_, while 10 and 15% increased total Chl and carotenoids (Table S2).

### 2.3. Relative Water Content (RWC)

**Figure 5:**
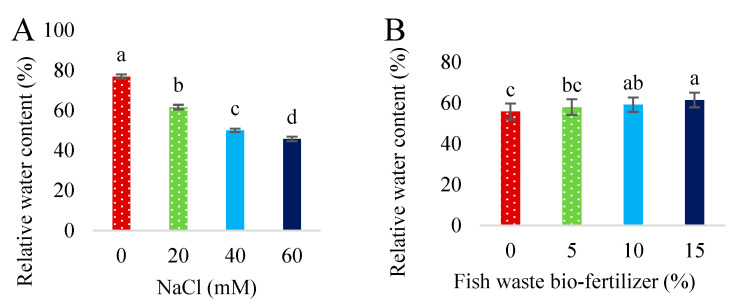
Effect of various concentrations of NaCl (**A**) and fish waste bio-fertilizer (**B**) on relative water content of *Stevia rebaudiana* Bertoni. Same letters indicate no significant differences (*p* < 0.05) based on Duncan’s Multiple Range test.

All salinity levels significantly (*p* < 0.05) reduced RWC compared with the control; the higher the salinity concentration, the lower the RWC. Salinity at 60 mM NaCl decreased RWC by 40.52% (Figure 5A). Fish waste bio-fertilizer at 10 and 15% significantly enhanced the RWC of stevia compared with the control (Figure 5B).

### 2.4. Total Carbohydrate Content

**Figure 6:**
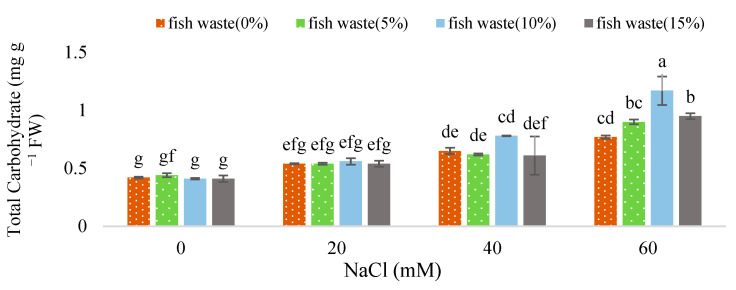
Effect of various concentrations of fish waste bio-fertilizer on total carbohydrates of *Stevia rebaudiana* Bertoni under salinity stress conditions (0, 20, 40, and 60 mM NaCl). Same letters indicate no significant differences (*p* < 0.05) based on Duncan’s Multiple Range test.

Salinity at 40 and 60 mM NaCl significantly enhanced total carbohydrate content compared with the control. Contrarily, the application of fish waste bio-fertilizer had no effects on the content under non-stress and 20 and 40 mM NaCl stress conditions. On the other hand, bio-fertilizer application significantly increased total carbohydrate content at 10 and 15% concentrations under 60 mM salinity stress (Figure 6).

### 2.5. Cellular Damage Indicators

**Figure 7:**
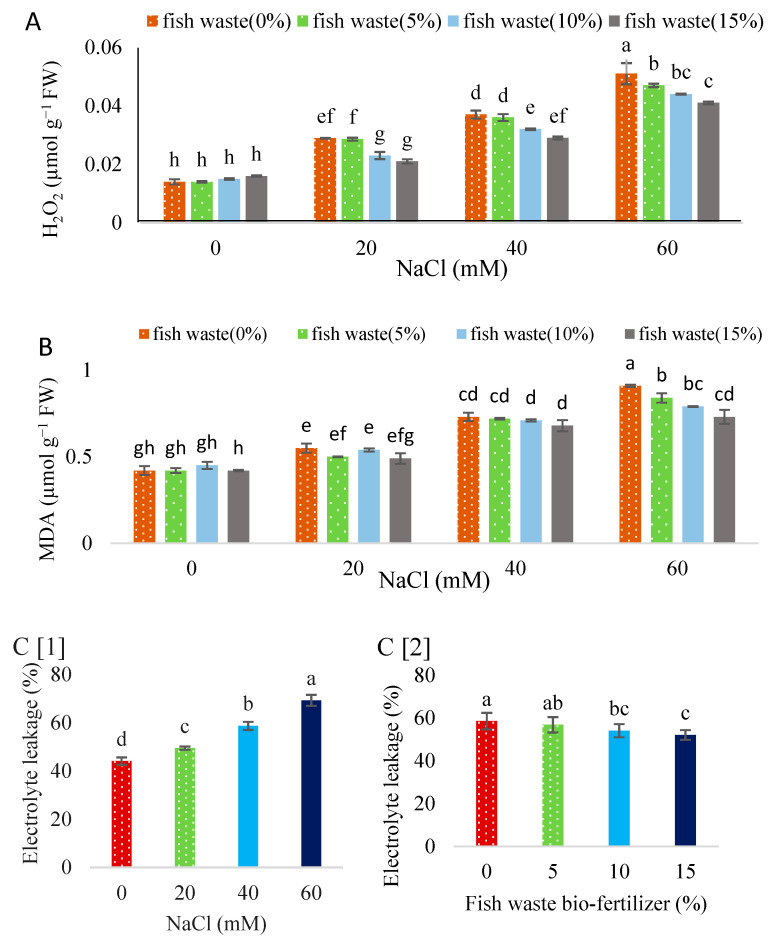
Effect of various concentrations of fish waste bio-fertilizer on (**A**) H_2_O_2_ and (**B**) MDA of Stevia rebaudiana Bertoni under salinity stress conditions (0, 20, 40, and 60 mM NaCl). Effect of various concentrations of NaCl (**C[1]**) and fish waste bio-fertilizer (**C[2]**) on electrolyte leakage of *Stevia rebaudiana* Bertoni. Same letters indicate no significant differences (*p* < 0.05) based on Duncan’s Multiple Range test.

Salinity at all applied concentrations caused significant (*p* < 0.05) enhancement in H_2_O_2_ (Figure 7A), MDA (Figure 7B), and electrolyte leakage (EL) (Figure 7C). The bio-fertilizer had no effect on H_2_O_2_ and MDA content under non-stress conditions, while 10 and 15% concentrations of fish waste bio-fertilizer led to a significant reduction in electrolyte leakage (EL). Under 20 and 40 mM NaCl, the bio-fertilizer applied at 10 and 15% concentrations significantly decreased H_2_O_2_, while no effect was observed in MDA content. Under 60 mM NaCl, all applied concentrations of the bio-fertilizer significantly lowered H_2_O_2_ and MDA content (Figure 7).

### 2.6. Proline and Total Phenolic Content

**Figure 8:**
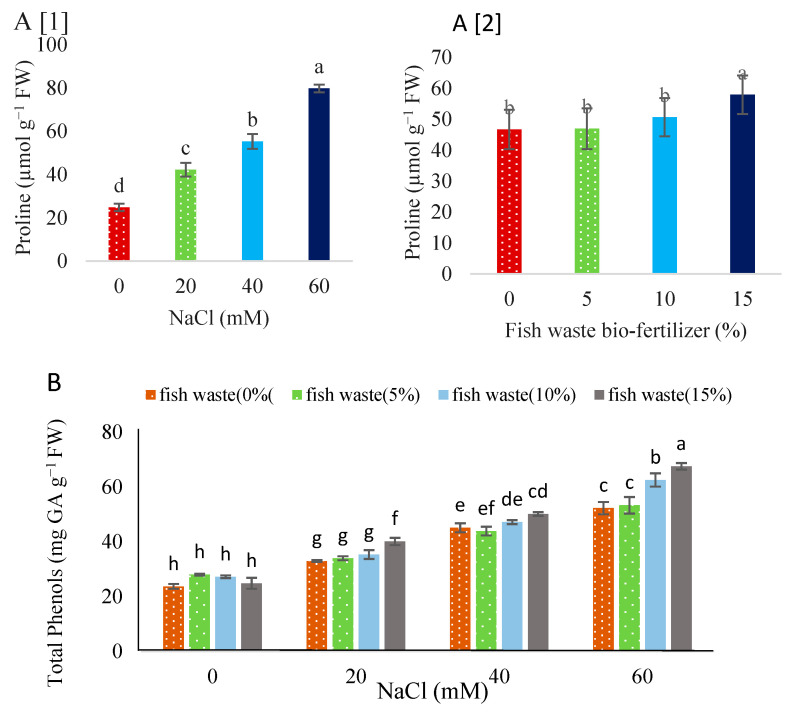
Effect of various concentrations of NaCl (**A[1]**) and fish waste bio-fertilizer (**A[2]**) on proline of *Stevia rebaudiana* Bertoni. Effect of various concentrations of fish waste bio-fertilizer on total phenols (**B**) of *Stevia rebaudiana* Bertoni under salinity stress conditions (0, 20, 40, and 60 mM NaCl). Same letters indicate no significant differences (*p* < 0.05) based on Duncan’s Multiple Range test.

Salinity, at all applied concentrations, increased proline (Figure 8A) and total phenols (Figure 8B) compared with the control. Salinity at 60 mM NaCl increased total phenols by 54.67% and proline by 68.88% compared to unstressed control. Fish waste bio-fertilizer had no effect on the phenol content under non-stress conditions, while a 15% concentration significantly enhanced proline content (Figure 7A). Under 20 and 40 mM NaCl, 15% bio-fertilizer enhanced total phenols, while both 10 and 15% concentrations caused an increase in total phenols under 60 mM NaCl (Figure 8).

### 2.7. Antioxidant Enzymatic Activities

**Figure 9:**
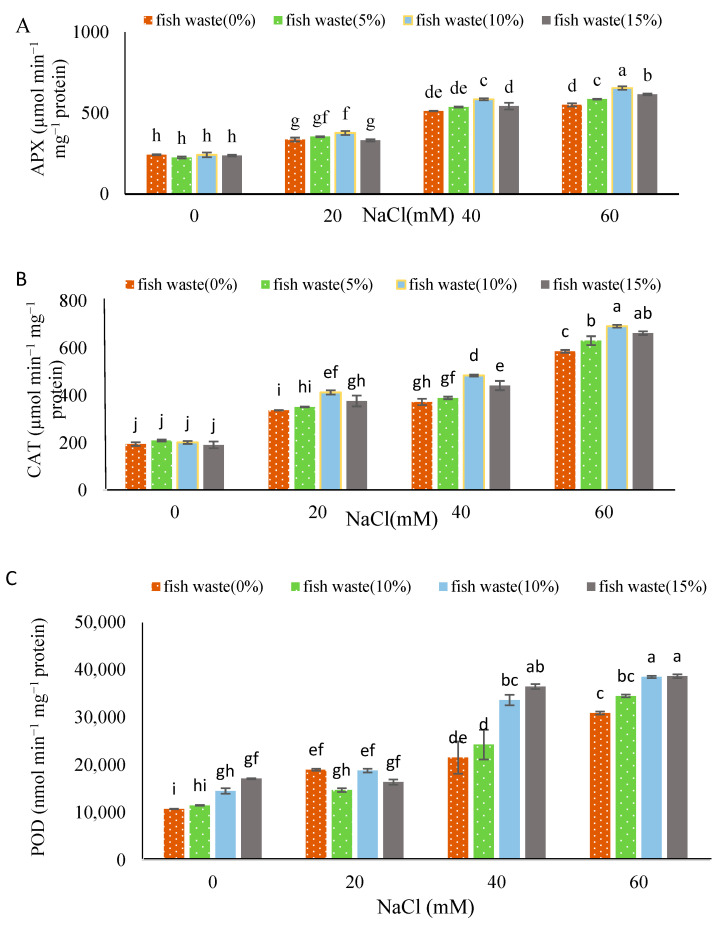
Effect of various concentrations of NaCl and fish waste bio-fertilizer on the enzymatic activity of (**A**) APX, (**B**) CAT, and (**C**) POD of *Stevia rebaudiana* Bertoni under salinity stress conditions (0, 20, 40, and 60 mM NaCl). Same letters indicate no significant differences (*p* < 0.05) based on Duncan’s Multiple Range test.

All salinity levels significantly (*p* < 0.05) enhanced enzymatic activities as compared with to the control (Figure 9). CAT activity in leaf tissues under all NaCl concentrations significantly increased compared to the untreated control. The maximum and minimum activities of CAT were recorded in 60 mM NaCl-treated plants under 10 and 15% fish waste bio-fertilizer concentration application and control samples, respectively (Figure 9B). In regard to APX, the enzymatic activity significantly increased under 20, 40, and 60 mM NaCl compared with the control. Therefore, increasing NaCl levels resulted in increasing APX activity. The highest activity among treatments was achieved at 10% fish waste bio-fertilizer concentrations under 60 mM NaCl, while the lowest was in all bio-fertilizer-treated plants under non-stress conditions (Figure 9A). The highest and lowest POD activities were observed in 10 and 15% bio-fertilizer-treated plants under 60 mM NaCl and non-stress conditions, respectively. Similar to APX and CAT, increasing salinity levels led to increasing POD activity, under no fish waste bio-fertilizer treatment. Fish waste bio-fertilizer treatments increased POD activity under both non-stress and stress conditions, with these increases being higher than non-treated plants at the same conditions (Figure 9C). Overall, fish waste bio-fertilizer at a 10% concentration generally increased antioxidant enzymatic activity under stress conditions compared with plants at similar conditions without receiving any bio-fertilizer treatments.

### 2.8. Elemental Composition of Shoots and Roots

**Figure 10:**
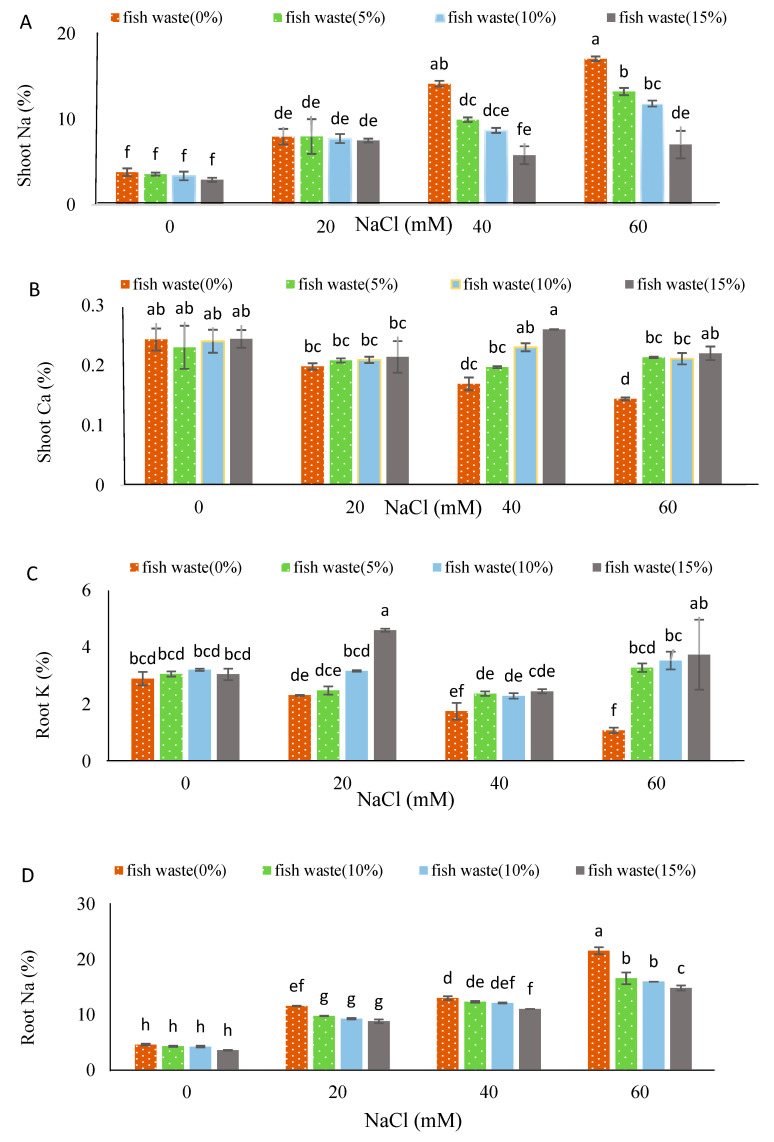
Effect of various concentrations of fish waste bio-fertilizer on essential nutrients: (**A**) shoot Na, (**B**) shoot Ca, (**C**) root K, and (**D**) root Na, of *Stevia rebaudiana* Bertoni under different concentrations of salinity stress (0, 20, 40, and 60 mM NaCl). Same letters indicate no significant differences (*p* < 0.05) based on Duncan’s Multiple Range test.

**Figure 11:**
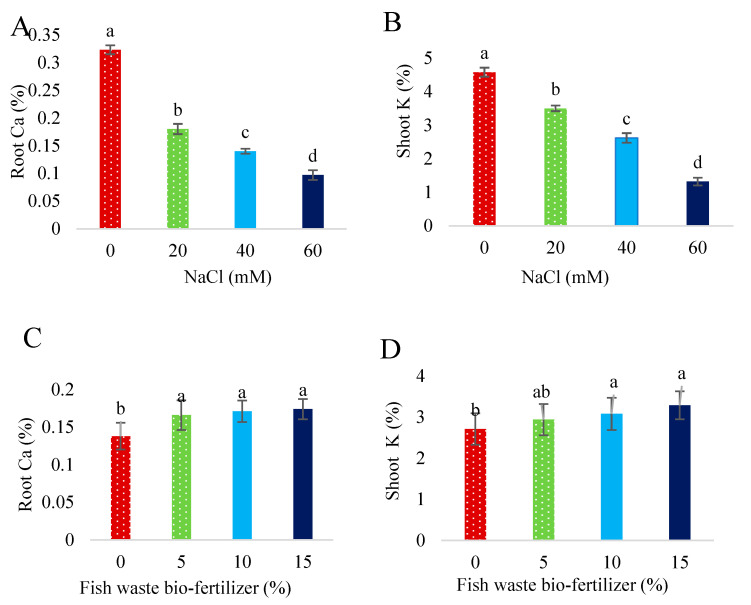
Effect of various concentrations of NaCl on (**A**) root Ca and (**B**) shoot K and effect of various concentrations of fish waste bio-fertilizer on (**C**) root Ca and (**D**) shoot K, of *Stevia rebaudiana* Bertoni. Same letters indicate no significant differences (*p* < 0.05) based on Duncan’s Multiple Range test.

Salinity stress increased the Na^+^ content of shoots and roots (Figure 10) and reduced the Ca^2+^ shoots and K^+^ roots contents (Figure 10), with increasing NaCl concentrations showing a positive correlation with Na^+^ content, and a negative correlation with Ca^2+^ and K^+^ contents, respectively. The bio-fertilizer had no effect on Na^+^ content in shoots and roots, Ca^2+^ content in shoots, or K^+^ content in roots (Figure 10). Salinity stress reduced the Ca^2+^ roots and K^+^ shoots contents (Figure 11). Whereas the bio-fertilizer enhanced Ca^2+^ content in roots at all concentrations and K^+^ content in shoots at 10 and 15% bio-fertilizer concentrations under non-stress conditions (Figure 11). Under 20 mM NaCl, the bio-fertilizer at all applied concentrations decreased Na^+^ content in roots, whereas K^+^ content increased in roots following an application at a 15% concentration. Under moderate salinity levels, fish waste bio-fertilizer decreased Na^+^ content in shoots at all applied concentrations, while application at a 15% concentration decreased Na^+^ content in roots and increased Ca^2+^ content in shoots (Figure 10). Under severe salinity levels, all concentrations of the bio-fertilizer reduced Na^+^ content in shoots and roots and enhanced Ca^2+^ content in shoots and K^+^ content in roots (Table S3).

### 2.9. Principal Component Analysis (PCA)

**Figure 12:**
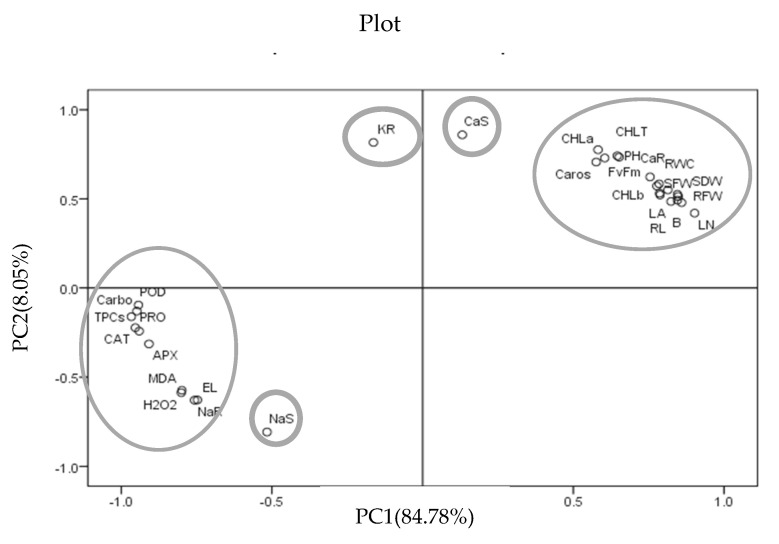
Plot from PCA performed on morphological, physiological, and biochemical traits in various concentrations of NaCl and fish waste bio-fertilizer of *Stevia rebaudiana* Bertoni. Abbreviations: root fresh weight (RFW), shoot fresh weight (SFW), shoot dry weight (SDW), root dry weight (RDW), relative water content (RWC), leaf number (LN), chlorophyll a (Chl_a), chlorophyll b (Chl_*b*), total chlorophyll (Chl_T), total carotenoids (Caros), Na^+^ concentration in shoots and roots (Na^+^ S; Na^+^ R), K^+^ concentration in shoots and roots (K^+^ S; K^+^ R), Ca^2+^ concentration in shoots and roots (Ca^2+^ S; Ca^2+^ R), proline (Pro), malondialdehyde (MDA), hydrogen peroxide (H_2_O_2_), total phenolic compounds (TPCs), Ascorbate peroxidase (APX), catalase (CAT), Peroxidase (POD), total carbohydrate (carbo), electrolyte leakage (EL), plant height (PH), root length (RL), leaf area (LA), and branches (B).

To demonstrate the negative impact of salinity stress in *Stevia rebaudiana* Bertoni plants with the application of fish waste bio-fertilizer, a principal component analysis was performed (Table 1, Figure 12). For the first two components, i.e., PC1 and PC2, cover was about 92.83% of the overall data base. Here, component PC1 contributes about 84.78%, correlating (biochemical and growth traits) significantly mainly with the activity of antioxidant enzymes (APX, POD, and CAT), total phenol content, carbohydrate, proline, malondialdehyde, chlorophyll b, relative water content, electrolyte leakage and growth traits (LN, SDW, RFW, B, LA, RDW, SFW, and RL), Na^+^ and Ca^2+^ contents in the roots, and K^+^ contents in the shoot. On the other hand, the second component, PC2, contributes 8.05%, correlating (photosynthetic pigments) mainly with the activity of chlorophyll a, total chlorophyll, carotenoids, ^Fv^/_Fm_, plant height, Na^+^ and Ca^2+^ contents in the shoots, and K^+^ contents in the root. Similarly, the principal component analysis revealed that different physiological and biochemical parameters correlated with different growth traits.

### 2.10. Correlation Analysis

Pearson’s correlation was performed among different photosynthetic pigments, ^Fv^/_Fm_ and the relative content of water and Na, K, and Ca in roots and shoots in the present work. Chl *a*, Chl *b*, total Chl, carotenoid content, RWC, and ^Fv^/_Fm_ content are positively correlated with K and Ca in roots and shoots and negatively correlated with Na in roots and shoots at a significant level (*p* < 0.05) (Table 2).

## 3. Discussion

In the current study, the effects of fish waste bio-fertilizer were investigated on the growth performance and physiological and biochemical characteristics of stevia plants subjected to salinity stress conditions.

According to the results, salinity stress mostly caused reduction in all morphological parameters whereas fish waste bio-fertilizer particularly at the 15% level increased these parameters, resulting in better plant growth. Salinity stress reduces photosynthesis and chlorophyll pigments, relative water content, and the absorption of important mineral ions including N and K, and simultaneously increases Na^+^ accumulation, all of which lead to a decrease in growth [21,22]. In fact, salinity has a negative effect on the growth and performance of plants by creating ionic toxicity, osmotic stress, and nutritional imbalance. In addition, salinity affects the availability, absorption, and transport of nutrients and water, and cell division, resulting in a decrease in growth previously confirmed in some plants [6,22,23,24], all in line with the current findings. Fish waste bio-fertilizer contains amino acids, organic acids, lactic acids, and acetic acids, all of which activate bacteria such as rhizobium and bacillus with the ability to dissolve P and stabilize N, Ca^2+^, K^+^, Na^+,^ Mg^2+^, Zn^2+^, and Mn^2+^ contents that then increase organic compounds and siderophore photosynthesis and chlorophyll pigments and synthesis for iron (Fe) sequestration. Increased access to the mentioned elements enhances photosynthesis, chlorophyll pigments, and plant growth, and, as a result, increases plant fresh weight [16]. Application of rural slaughterhouse waste, as a bio-fertilizer, improved plants growth when applied after planting (at two and six weeks) [25]. Fish waste bio-fertilizer causes the release of fulvic acid and humic acid, the decomposition of which creates auxin, as well as the increase in P and K and other nutrient absorption. The application of fish waste bio-fertilizer increased the growth of red chili and tomato plants [26], and the root length of *Solanum melongena* [16], onion [27], and *Prunus persica* [28], in line with the current results. Salinity reduced the amount of photosynthetic pigments and ^Fv^/_Fm_ in stevia plants, while fish waste bio-fertilizer improved their amounts probably via preventing pigment decomposition. The chlorophyll fluorescence index is one of the most important factors to evaluate photosynthetic efficiency due to its sensitivity to early plant responses to stress conditions. According to our findings, a decrease in the ^Fv^/_Fm_ index was reported in sweet pepper and wheat under salinity stress [28]. Salinity damages chloroplast membranes and the electron transport chain in photosystem I, and reduces the absorption of Mg and K, resulting in negative feedback on chlorophyll fluorescence parameters [29]. Salinity-induced Cl^-^ ions have destructive impacts on the reaction center of photosystem II (PSII), the quinone receptor, and the oxygen transport system. This destructive impacts results in a decrease in light energy of the reaction center and photosynthetic pigments and finally decrease photosynthesis process. Salinity-induced Cl^-^ ions have destructive impacts on the reaction center of photosystem II (PSII), the quinone receptor, and the oxygen transport system, which then results in a decrease in light energy of the reaction center and photosynthetic pigments and process. Salinity causes osmotic tension via producing reactive oxygen species (ROS) through causing a high concentration of Na^+^ in plant tissues that decreases CO_2_ and finally photosynthetic processes. Moreover, salinity increases the activity of chlorophyllase, the enzyme involved in the decomposition of photosynthetic pigments [30] and damages chloroplasts [31].

There was a negative correlation between the increase of Na^+^ ions and the amount of photosynthetic pigments. Lower chl content during salinity stress was identified as a good indicator of the sensitivity of stevia plants to salinity conditions because it connotes damage in the chloroplast membranes and oxygen transport system, and finally a reduction in photosynthesis [32]. The negative effect of salinity on photosynthetic pigments and processes was previously confirmed in several plant species [22,24,30,33].

There is a positive correlation between the increase in Ca^2+^ and K^+^ ions and the amount of photosynthetic pigments. Higher chl content during the application of fish waste bio-fertilizer was identified as a good indicator of the fact that the application of the bio-fertilizer is effective under salinity conditions. Ca enhances the number of leaves and maintains the pH of the cell for electron transports in chloroplast membranes, and finally maintains photosynthesis and reduces oxygen species (ROS). K causes an increase in the carbon assimilation rate and chl metabolism, and increases photosynthesis rate [34]. In line with the current findings, the positive effect of K and Ca on photosynthetic pigments was reported in Wheat [34].

Fish waste bio-fertilizer enhances the production of antioxidants, vitamins, proteins, and alkaloids in plants thanks to its amino acid content such as glycine and glutamine, as precursors for Chl biosynthesis [35]. Moreover, this bio-fertilizer enhances nutrient absorption, especially P [26], and metabolic processes of the plant that result in increased leaf surface area and finally photosynthetic pigments and photosynthesis. As mentioned, the bio-fertilizer is rich in Mg and N, two main elements of Chl [16]. The amino acid glutamate in fish waste bio-fertilizer increases vegetative growth by increasing the synthesis of chlorophyll and other amino acids (aspartic acid, serine, alanine, lysine, and proline). This amino acid increased vegetative growth in soybean plant [36]. The amino acid alanine found in fish waste bio-fertilizer has a dual function between carbon and nitrogen metabolism, and, finally, this amino acid is associated with increasing chlorophyll synthesis and photosynthesis [37]. The amino acid glutamine in fish waste fertilizer increases nitrogen uptake in rice plants under cadmium stress by increasing plant growth regulators [38]. Therefore, fish waste bio-fertilizer enhanced the photosynthetic pigments and processes thanks to the mentioned reasons formerly confirmed [16,39]. That could be observed under salinity conditions. In line with the current findings, the positive effect of fish waste bio-fertilizer was reported on the leaf area and photosynthetic pigment biosynthesis of pumpkin [40] and cucumber [40] plants.

Salinity led to a reduction in RWC due to an imbalance in ions and osmotic pressure. In addition, salinity caused an enhancement in proline and sugar. On the other hand, the bio-fertilizer improved RWC as well as the proline and sugar content of stevia plants. Plant relative water content (RWC) is a vital physiological indicator in plants. Salinity stress disturbs the reasonable absorption of water and concomitantly osmotic equilibrium in the root zone, and, as a result, plant RWC decreased as previously reported [41,42]. The fish waste bio-fertilizer has a high K^+^ content, an element with positive impacts on the osmotic potential and root growth that leads to increased water absorption [43]. The amino acids glycine and proline in fish waste bio-fertilizer increase the osmotic potential of plant cells, thereby leading to increased water absorption [44], in line with the current findings.

Plants commonly enhance the release of compatible solutes such as proline and sugars in response to stress-induced reduced osmotic potential and cellular water [45]. Proline is an osmolyte capable of neutralizing ROS over stress conditions. Proline and carbohydrates increase the activity of the Rubisco enzyme, the primary carboxylase of photosynthesis which then protects photosynthesis, cell expansion, membrane stability and flexibility, and plant growth under stress conditions [21]. Moreover, the biosynthesis pathways of proline and sugar are closely tied together due to glutamate production, and an increase in proline biosynthesis leads to sugar production and vice versa. Salinity caused an increase in sugar and proline in some plants [22,24,46]. Mostly, an increase in proline synthesis and a decrease in its oxidation are the reasons for proline accumulation under salinity stress [47]. A high concentration of Na^+^ reduces the absorption of Ca^2+^, Mg^2+^, and K^+^, leading to an enhanced Na^+^/K^+^ ratio. On the contrary, fish waste bio-fertilizer has a high K^+^ content that results in a reduced Na^+^/K^+^ ratio and then better stomatal performance. As a result, stomata open and CO_2_ diffusion to plant tissues increases, and then the photosynthesis rate is enhanced, which finally leads to carbohydrate (sugar) production [43], as previously confirmed in spinach plants [19]. In addition, the amino acid leucine in fish waste bio-fertilizer acts as a precursor for alanine synthesis in plants. In plants, alanine is important for the synthesis of pantothenate and subsequently coenzyme A, an essential coenzyme in carbohydrates. The carbohydrate produced in the plant plays a key role in the opening and closing of the stomata [48]. An increase in carbohydrate production by using the amino acids leucine and alanine has been reported in wheat plants [48], in line with the current results.

The enhanced amount of proline after the bio-fertilizer application referred to its high N and P content that helped mitigating the salinity effects [49]. In addition, fish waste fertilizer contains proline, which acts as an osmotic protector in abiotic stress and also as a metal chelator [36]. Proline and glutamine make the tomato plant resistant to salt stress [44], and all mentioned studies and explanations are in accordance with the current findings.

Salinity increased the total phenols and the bio-fertilizer increased the content even more in the stevia plant. Under stress conditions, plants activate enzymatic and non-enzymatic antioxidant systems to lessen the stress impacts. Phenolics are non-enzymatic antioxidants whose amounts are enhanced under salinity to neutralize ROS and prevent the decomposition of hydroperoxide [50]. An increase in phenols was previously reported in some plants under salinity conditions [51,52], as observed in the current study. Fish waste bio-fertilizer contains some phenol- and flavonoid-based compounds that could stimulate phenolic content in the plant [53]. Also, the amino acid phenylalanine in fish waste fertilizer increases the phenolic compounds of the plant and increases the antioxidant capacity through the shikimic acid pathway. The production of phenolic compounds from the amino acid phenylalanine has been observed in grape plants [54], in line with the current findings. Salinity increased electrolyte leakage (EL), MDA, and H_2_O_2_ and the application of fish waste bio-fertilizer reduced their amounts. Normally, plant cells produce small amounts of H_2_O_2_ and this creates a defense mechanism in the plant. Salinity causes oxidative stress, leading to ROS and H_2_O_2_ high production and accumulation which then damages membranes and enhances electrolyte leakage (EL) and MDA [22,24]. An increase in MDA demonstrated the plant’s incapability to remove ROS and H_2_O_2_ since ROS and H_2_O_2_ accumulation led to the peroxidation of fats, the deactivation of enzymes, damage to nucleic acids, and the destruction of cell membranes. Salinity caused the conversion of superoxide radical (O_2_^−^) to H_2_O_2_ inside the cell, which hinders the Calvin cycle and finally the activity of antioxidant enzymes (e.g., CAT and SOD). Next, H_2_O_2_ prevents sugar biosynthesis in chloroplasts [45]. Salinity increased MDA content in soybean [55] and stevia [2] and H_2_O_2_ content in basil [56] and wheat [57]. An increase in H_2_O_2_ resulted in enhanced electrolyte leakage (EL) [24,58]. An increase in salinity resulted in enhanced MDA, which was additionally confirmed in sweet basil [59,60]. P content in the bio-fertilizer increases the production of phospholipids that strengthen the cell membranes and thus reduce MDA and H_2_O_2_ [26]. Also, glycine and proline in fish waste fertilizer increase the stability of the two layers of the plasma membrane by increasing the activity of catalase and superoxide dismutase enzymes and regulating the peroxidation of lipids and homeostasis of ions. Glycine treatment increased salinity tolerance in safflower, reduced MDA and H_2_O_2_, and improved homeostasis [61], which could explain the current results. Salinity enhanced antioxidant enzymes (POD, CAT, and APX) and fish waste bio-fertilizer increased their activities further. Under salinity conditions, plants increase several antioxidant enzymes’ activities (e.g., POD, CAT, and APX) to neutralize salinity-induced ROS effects [62], as was formerly confirmed [22,24,63,64]. The nutrient content of the bio-fertilizer, especially C, N, and P, as well as the amino acids, increased the antioxidant enzymes’ activities [26] like CAT and POD in lettuce and cowpea [13,39]. N present in fish waste bio-fertilizer is used as a precursor for the production of protein and antioxidant enzymes under salinity conditions. Arginine, as an amino acid in the bio-fertilizer, is involved in salinity stress tolerance by producing spermidine (a polyamine) or nitrate reductase (reducing nitrate to nitrite) [65]. Different amino acids, such as the amino acid proline found in fish waste bio-fertilizer, play a key role in the antioxidant defense system and the production of various enzymes in stressful conditions [36]. The positive effects of fish waste bio-fertilizer have been proven in this regard [66], all in line with the current results.

The current results demonstrated that salinity increased Na^+^ and reduced K^+^ and Ca^2+^ contents of stevia plants, as previously confirmed [1,28,41,67]; on the contrary, the application of fish waste bio-fertilizer reduced Na^+^ and increased K^+^ and Ca^2+^ contents. Under salinity, H^+^-ATPases of the plasma membrane create a H^+^ gradient that provides the necessary energy for the secretion of K^+^ via the H^+^/K^+^ antiport and thereby ensures Na re-absorption [68]. Salinity additionally causes osmotic pressure in the rhizosphere soil solution that, as a result, decreases the uptake of water and minerals, (e.g., K^+^ and Ca^2+^) due to the antagonistic effect [69]. It seems that the bio-fertilizer reduced Na^+^ accumulation in the aerial parts through Na^+^ removal from the xylem [70]. Thanks to the high solubilization and availability of the Ca^2+^ and K^+^ contents of the fish waste bio-fertilizer, its application could compensate for the lack of these elements caused by salinity [15]. The bio-fertilizer application increased N, P, K, and Ca in spinach [19], in line with the current results.

The PCA analysis showed that the measured traits were divided into two principal components. The first one, PC1, included biochemical and morphological traits and the second principal component, PC2, contained physiological traits. The plot also showed a high correlation between physiological and morphological traits.

PCA was also identified for the assessment of stress conditions in crop species such as wheat [71]. These principal components were most likely to have the most effect on the overall variation of the data set and could be identified as significant contributors to the salinity response in various plants. In other words, these components demonstrated the strongest association with salinity stress and the modulator of stress. These statistical tools allow the identification of probable components and associations among accessions and traits [71].

## 4. Materials and Methods

This study was conducted in the research greenhouse of the Faculty of Agriculture, Mohaghegh Ardabili University (38 2514′ N; 48), in a factorial experiment, and was based on a completely randomized design with three replications in the spring-summer of 2021. The first factor was salinity stress at four levels (0, 20, 40, and 60 mM NaCl) and the second factor was liquid fish waste bio-fertilizer (foliar application) at four levels (0, 5, 10, and 15% (*v*/*v*)). The fish waste of *Hypophthalmichthys molitrix* (head, tail, and fin) was purchased from local fishmongers in Ardabil city. After washing, the waste was dried in the shade and then ground, while the resulting powder was autoclaved for 20 min. After mixing the dry material with distilled water at a ratio of 1:5, 30 mL protein-hydrolyzing bacteria (*Bacillus subtilis*) and 150 g sugar were added and incubated at 25 °C for two weeks according to [72]. Finally, membrane filters (Filtration-Micro MF) were used for separating the liquid phase, and then free amino acids (Table 3) as well as an elemental analysis (Table 4) of the total liquid fish bio-fertilizer were measured using HPLC.

Stevia seedlings (*Stevia rebaudiana* Bertoni) were purchased from a medicinal plant greenhouse in Shiraz city and planted in 10 L pots containing a mixture of cocopeat and perlite (1:1). The plants were first irrigated with half-strength Hoagland’s solution (4 weeks) and then irrigated with full-strength nutrient solution (up to harvest: 400 mL every day). The hydroponic solution contained the following macronutrients (mg/L): nitrate (N) 210, potassium (K) 204, calcium (Ca) 140, sulphur (S) 64, magnesium (Mg) 48, phosphorus (P) 31, and micronutrients (mg/L): iron (Fe) 4, boron (B) 0.5, manganese (Mn) 0.5, copper (Cu) 0.1, zinc (Zn) 0.1, and molybdenum (Mo) 0.05. The half-strength Hoagland’s solution had a conductivity of 1.022 dS/m. The pH of the Hoagland’s solution was sustained at 5.97 throughout the experiment. Following plant establishment (two weeks after the transferred seedlings to pots), salinity was applied by adding the mentioned concentrations of NaCl (0, 20, 40, and 60 mM) to the nutrient solution. The plants were first irrigated with half NaCl concentration (7 days) and then irrigated with the desired NaCl concentrations (0, 20, 40, and 60 mM) until harvesting the plant. After the initiation of salinity treatments, plants were grown for 8 weeks. The leaves (aerial parts) were sprayed with the mentioned concentrations of fish waste bio-fertilizer (0, 5, 10, and 15% along with Tween 0.1%) two weeks after salinity application 4 times, with an 8-day interval. The control plants were irrigated with the nutrient solution in the same manner up to the harvest and treated with any treatments. The plants were harvested 80 days after planting and samplings were performed through the leaves. Each measurement was performed in triplicate.

### 4.1. Morphological Parameters

Shoot height, root length, fresh and dry weights of roots and shoots, number of leaves, branches, and leaf area were recorded as morphological parameters. To measure dry weight, the plant materials were kept at 70 °C for 72 h in a drying oven. Ten leaves were selected of random from each replicate to measure leaf area using Pamwin software (PAM 2500).

### 4.2. Photosynthetic Pigments and Chlorophyll Fluorescence Parameter

Photosynthetic pigments and carotenoids were measured according to the method described in [73]. For this purpose, 0.1 g of fresh leaves was homogenized with 5 mL of acetone (80%) and then the obtained mixture extract was centrifuged at 10,000 rpm for 10 min. Using a UV-V device (Hitachi U-2910, Tokyo, Japan), the absorption of the samples was recorded at wavelengths of 470, 646.8, and 663.2 nm to determine carotenoids and chlorophyll (Chl a and Chl b), respectively, based on the following formulas:

Chl a = (19.3 × A663.2 − 0.86 × A646.8) [V/100 × W]

Chl b = (19.3 × A646.8 − 3.6 × A663.2) [V/100 × W]

Carotenoids = [100(A470) − 3.27 (chl b)]/227

Chl_Total_ = Chl a + Chl b

Where V is the volume of the extract and W is the weight of fresh material.

The chlorophyll fluorescence parameter (^Fv^/_Fm_) was measured using a fluorometer (model OS-30p) after a 20 min adaption of leaves in the dark.

### 4.3. Relative Water Content (RWC)

Relative water content was measured according to the method described in [74]. For this purpose, three young and developed leaves from each pot were weighed (FW) after harvesting. The leaves were then placed in water for 24 h, in darkness and in a refrigerator; then, turgid weight (TW) was measured. At that time, the leaves were dried in an oven at 70 °C for 48 h inside paper envelopes to obtain their dry weight (DW). Relative water content was calculated using the following formula:

RWC% = [(FW − DW)/(TW − DW)] × 100

### 4.4. Soluble Carbohydrates

The amount of soluble carbohydrates was measured using anthrone reagent [75]. For this purpose, 0.1 g of fresh leaves was homogenized with 5 mL ethanol (80%) and placed in a hot water bath (95 °C, 10 min). The resulting suspension was centrifuged at 10,000 rpm for 10 min. Then, 3 mL of anthrone reagent was added to 100 µL of prepared extract and the absorbance was recorded at 620 nm using a UV-V spectrophotometer (Hitachi U-2910, Tokyo, Japan). Finally, glucose concentration was determined using a standard curve.

### 4.5. Total Protein Content

To assay the concentration of protein in stevia extracts, the wet leaves of samples were homogenized by 3 mL of sodium phosphate buffer (pH = 6.8). The samples were centrifuged at 13,000 rpm for 15 min. The reaction mixture contained 100 μL of enzyme extract and 900 μL of Bradford reagent. The reaction was measured at a wavelength of 595 nm [76].

### 4.6. Hydrogen Peroxide (H_2_O_2_)

H_2_O_2_ content was determined first by homogenizing leaf tissues (0.1 g fresh weight) in 2 mL of trichloroacetic acid (TCA, 0.1% (*w*/*v*)). Then, 500 μL of the extract was added to the reaction mixture containing 500 μL of phosphate buffer (100 mM, pH = 7) and 2 mL of potassium iodide (1 mM). Lastly, the absorbance was measured at 620 nm using a spectrophotometer [77].

### 4.7. Malondialdehyde (MDA)

To measure MDA, 0.5 g of fresh leaves was homogenized in 6 mL of trichloroacetic acid (1% (*w*/*v*)) and then centrifuged. Next, the supernatant was separated and 2 mL of thiobarbiotic acid solution was added and then centrifuged (10,000 rpm, 10 min). The absorption was recorded at 532 nm and 600 nm and converted to the exact amount [78].

### 4.8. Electrolyte Leakage (EL)

The method of [79] was used to measure electrolyte leakage (EL). Based on this method, EL was determined using an electrical conductivity meter (Hanna, HI98192, Hanna Instruments, Inc., Woonsocket, RI, USA). The initial electrical conductivity (EC1) was recorded after washing the discs (0.5 cm diameter) of leaves three times with deionized water and incubating them at ambient temperature (24 h). The final electrical conductivity (EC2) was measured after incubating the samples in a water bath (95 °C, 20 min) and cooling down the samples at 25 °C. Lastly, electrolyte leakage EL was calculated from the following equation.

*EL* (%) = (*EC*1/*EC*2) × 100

### 4.9. Proline Content

To measure proline content, 0.1 g of fresh leaf tissues was homogenized in 5 mL of sulfosalicylic acid) 3% (*w*/*v*)) and centrifuged. The reaction mixture included 2 mL of extract, 2 mL of ninhydrin acid, 2 mL of acetic acid, and toluene. The absorbance was measured at 520 nm using a spectrophotometer (Hitachi U-2910, Tokyo, Japan) [80].

### 4.10. Total Phenolic Content

Phenolic content was determined through homogenizing leaf tissues (0.2 g of fresh weight) in 2 mL of ethanol (70%), kept for 24 h in the dark at 4 °C. Then, to 500 µL of extract, 500 µL of ethanol (96%) and distilled water (1.5 mL) were added; then, Folin–Ciocalteu reagent and sodium carbonate were mixed with the mixture. Finally, the absorbance was measured at 725 nm with the spectrophotometer [81].

### 4.11. Activity of Antioxidant Enzymes

After homogenizing leaves (0.5 g) with potassium phosphate buffer (pH 6.8, 10 mM), the extract was centrifuged (6000 rpm, 20 min) and the supernatant was used for the assay of the enzymatic activities.

#### 4.11.1. Peroxidase Enzyme (POD)

To assay POD activity, the reaction mixture contained 2.9 mL of sodium phosphate buffer (100 mM), guaiacol (100 mM), and 50 μL of enzyme extract. The reaction was initiated at a wavelength of 470 nm by the addition of H_2_O_2_ (20 mM) after 60 s. A blank sample was prepared without enzyme extract [82].

#### 4.11.2. Catalase (CAT)

CAT activity was measured according to the method described in [83]. The reaction mixture consisted of 2.5 mL of potassium phosphate buffer (100 mM, pH = 7), H_2_O_2_ (5 mM), and 60 μL of enzyme extract. The reduction in absorbance was measured by the degradation of H_2_O_2_ at 240 nm using the spectrophotometer.

#### 4.11.3. Ascorbate peroxidase (APX)

The reaction mixture for the APX assay consisted of potassium phosphate buffer (50 mM, pH = 7), 100 μL of enzyme extract, ascorbic acid (50 μM), and H_2_O_2_ (1.5 mM), and the absorbance was recorded at 290 nm using the spectrophotometer [84].

### 4.12. Sodium (Na), Potassium (K), and Calcium (Ca) Content of Shoots and Roots

To measure the amount of macronutrients (potassium, K; calcium, Ca; and sodium, Na), stevia shoots and roots were ashed in an oven at 550 ± 25 °C. White ash was digested in 10 mL of concentrated hydrochloric acid (HCl) and brought up to a volume of 100 mL for the measurement of Na^+^, potassium (K), and calcium (Ca). Sodium and potassium concentrations were quantified using a flame photometer. Ca concentration was recorded with an atomic absorption instrument [85].

### 4.13. Statistical Analysis

Data were analyzed using SAS 9.1 software by analyzing the means using Duncan’s multiple range test, with a significant difference level at *p* < 0.05.

## 5. Conclusions

In spite of the fact that fish waste can be used as a bio-fertilizer, little research has been conducted on its use to lessen the effects of various stress conditions. Accordingly, the current study aimed to shed light on the application of fish waste as a bio-fertilizer on stevia plants under salinity stress. The bio-fertilizer increased morphological parameters, photosynthetic pigments, ^Fv^/_Fm_, RWC, proline, phenols, and antioxidants, and reduced sugar, EL, MDA, and H_2_O_2_. In fact, the presence of different amino acids and nutritional elements in fish waste bio-fertilizer caused an enhancement in antioxidant enzyme activities and a reduction in the salinity-induced oxidative damages in stevia plants. The bio-fertilizer, particularly at a 15% concentration, could be introduced as the best dose based on the measured parameters to lessen salinity effects. Hence, fish waste bio-fertilizer could be considered an effective bio-fertilizer to apply on plants under different stress conditions to mitigate stress effects through a safe and environmentally friendly method. In addition, fish waste bio-fertilizer did not cause more salinity issues, at least with the applied doses and experiment time, which is an imperative aspect.

## Figures and Tables

**Table 1:** Principal components analysis for morphological, physiological, and biochemical traits of *Stevia rebaudiana* Bertoni under salinity stress conditions.

Traits	First Principal Components	Second Principal Components
Total phenol content (TPCs)	−0.967	-
Catalase (CAT)	−0.953	-
Carbohydrate (Carbo)	−0.948	-
Peroxidase (POD)	−0.943	-
Proline (PRO)	−0.941	-
Ascorbate peroxidase (APX)	−0.908	−0.314
Leaf number (LN)	0.903	0.420
Shoot dry weight (SDW)	0.860	0.479
Root fresh weight (RFW)	0.848	0.513
Shoot K (KS)	0.845	0.525
Branches (B)	0.845	0.493
Leaf area (LA)	0.824	0.485
Root dry weight (RDW)	0.814	0.550
Hydrogen peroxide (H_2_O_2_)	−0.801	−0.587
Malondialdehyde (MDA)	−0.799	−0.574
Shoot fresh weight (SFW)	0.788	0.522
Root length (RL)	0.786	0.532
Chlorophyll b (ChLb)	0.785	0.584
Relative water content (RWC)	0.777	0.572
Root Na (NaR)	−0.757	−0.629
Root Ca (CaR)	0.755	0.623
Electrolyte leakage (EL)	−0.747	−0.628
Shoot Ca (CaS)	-	0.859
Root K (KR)	-	0.816
Shoot Na (NaS)	−0.516	−0.808
Chlorophyll a (ChLa)	0.582	0.776
Total chlorophyll (ChLT)	0.646	0.741
Plant height (PH)	0.652	0.734
^Fv^/_Fm_	0.604	0.729
Carotenoids (Caros)	0.576	0.707
Percentage of variance	840.78%	80.05%

**Table 2:** Pearson correlation coefficients (r) between photosynthetic pigments, ^Fv^/_Fm_ and the relative content of water and sodium, potassium, and calcium in roots and shoots of *Stevia rebaudiana* Bertoni under salinity stress conditions (* *p* < 0.05; ** *p* < 0.01).

	^Fv^/_Fm_	Chl a	Chl b	Total Chl	Carotenoids	RWC	Shoot Na	Shoot K	Shoot Ca	Root Na	Root K	Root Ca
^Fv^/_Fm_	1											
Chla	0.927 **	1										
Chlb	0.916 **	0.898 **	1									
Total Chl	0.942 **	0.993 **	0.942 **	1								
Carotenoids	0.858 **	0.856 **	0.865 **	0.857 **	1							
RWC	0.847 **	0.929 **	0.49 **	0.95 **	0.883 **	1						
Shoot Na	−0.883 **	−0.948 **	−0.849 **	−0.942 **	−.846 **	−0.859 **	1					
Shoot K	0.901 **	0.905 **	0.964 **	0.938 *	0.843 **	0.951 **	−0.864 **	1				
Shoot Ca	0.706 **	0.769 **	0.587 *	0.737 **	0.518 *	0.556 *	−.839 **	0.577 **	1			
Root Na	−0.931 **	−0.939 **	−0.960 **	−0.963 **	−0.861 **	−0.949 **	0.904 **	−0.971 **	−0.673 **	1		
Root K	0.457	0.48	0.398	0.468	0.562 *	0.371	−0.464	0.27	0.488	−0.363	1	
Root Ca	0.897 **	0.933 **	0.957 **	0.957 **	0.864 **	0.957 **	−0.837 **	0.967 **	0.616 **	−0.97 **	0.408	1

**Table 3:** Composition of amino acids in fish waste bio-fertilizer. Content shown as mg Amino Acid (AA)/g sample.

Amino Acid	Free Amino Acid	Total Amino Acid	Unit (mg Amino Acid(AA)/g Sample)
Asp	0.7	1.36	mg AA/g sample
Glu	1.49	3.57	mg AA/g sample
Ser	0.17	0.87	mg AA/g sample
Gly	1.08	3.42	mg AA/g sample
His	0.15	0.48	mg AA/g sample
Arg	0.23	1.02	mg AA/g sample
Thr	0.14	0.84	mg AA/g sample
Ala	1.39	2.77	mg AA/g sample
Pro	0.61	1.86	mg AA/g sample
Tyr	0.48	0.48	mg AA/g sample
Val	0.72	1.63	mg AA/g sample
Met	0.26	0.51	mg AA/g sample
(cys)2	0.00	0.00	mg AA/g sample
Ile	0.47	0.94	mg AA/g sample
Leu	0.98	1.7	mg AA/g sample
Phe	0.41	0.83	mg AA/g sample
Lys	0.28	0.92	mg AA/g sample

**Table 4:** The composition of major nutrients of fish waste bio-fertilizer.

Ca%	P%	K%	N_total_%	OC (Organic Carbon)%
1.4	4.1	6.23	5.92	12.5

## Data Availability

Data will be made available upon request.

## Supplementary Materials

The following supporting information can be downloaded at: https://www.mdpi.com/article/10.3390/plants13141909/s1, Table S1: Mean comparisons of salinity stress and fish waste bio-fertilizer on morphological traits of *Stevia rebaudiana* Bertoni. Same letters indicate no significant differences (*p* < 0.05) based on Duncan’s test; Table S2: Mean comparisons of salinity stress and fish waste bio-fertilizer on proline, electrolyte leakage (EL) and relative water content (RWC) of *Stevia rebaudiana* Bertoni. Same letters indicate no significant differences (*p* < 0.05) based on Duncan’s test; Table S3: Effect of salinity stress and fish waste bio-fertilizer on shoot potassium (K) and root calcium (Ca) of *Stevia rebaudiana* Bertoni. Same letters indicate no significant differences (*p* < 0.05) based on Duncan’s test.
