# Supplementary material for: Fish Waste—A Novel Bio-Fertilizer for Stevia (Stevia rebaudiana Bertoni) under Salinity-Induced Stress
Source: Plants (Basel). 2024 Jul 11;13(14):1909. doi: 10.3390/plants13141909 (PMC11280417; doi:10.3390/plants13141909)
Supplement: Supplementary file 1 [file plants-13-01909-s001.zip › plants-3066266-supplementary.pdf]

Table S1.Mean comparisons of salinity stress and fish waste bio-fertilizer on morphological traits of *Stevia rebaudiana* Bertoni. The same letters do not differ significantly ($p < 0.05$) based on Duncan 's test.

Treatment	Concentration	Root length (cm)	Root DW (g)	Root FW (g)	Leaf area (mm^2)	Number of leaves	branches	Shoot DW (g)	Shoot FW (g)
NaCLmM)	0	27.25[a]	10.94[a]	63.45[a]	55.85[a]	640.08[a]	44.16[a]	31.58[a]	209[a]
	20	25.83[a]	9.6[b]	50.27[b]	55.65[a]	506.5[b]	36.83[b]	23.06[b]	181.92[b]
	40	21.66[b]	8.27[c]	40.86[c]	38.91[b]	355[c]	29.41[c]	17.69[c]	164.06[c]
	60	13[c]	6.78[d]	29.97[d]	28.55[c]	185.42[d]	16.33[d]	10.26[d]	106.8[d]
Fish waste biofertilizer (%)	0	20[b]	8.69[a]	44.19[a]	40.88[b]	411.33[a]	30.25[b]	18.89[b]	155.205[b]
	5	21.77[ab]	8.76[a]	44.85[a]	43.79[ab]	424[a]	29.75[b]	20.48[ab]	159.099[b]
	10	22.66[a]	8.95[a]	47.09[a]	45.49[a]	429.83[a]	32.25[ab]	21.15[ab]	169.84[ab]
	15	23.33[a]	9.19[a]	48.42[a]	46.8[a]	421.83[a]	34.5[a]	22.08[a]	181.612[a]

Table S2. Mean comparisons of salinity stress and fish waste bio-fertilizer on proline, electrolyte leakage (EL) and relative water content (RWC) of *Stevia rebaudiana* Bertoni. The same letters do not differ significantly ($P < 0.05$) based on Duncan 's teste.

Treatment	Concentration	Proline (µmol g^{-1} FW)	Electrolyte leakage (%)	Relative water content (%)
NaCl(mM)	0	24.84[d]	44.09[d]	76.912[a]
	20	42.26[c]	49.47[c]	61.61[b]
	40	55.32[b]	58.75[b]	50.006[c]
	60	79.83[a]	69.34[a]	45.74[d]
Fish waste biofertilizer (%)	0	46.71[b]	58.59[a]	55.76[c]
	5	46.96[b]	56.84[ab]	57.95[bc]
	10	50.664[b]	54.11[bc]	59.124[ab]
	15	57.946[a]	52.109[c]	61.43[a]

Table S3. Effect of salinity stress and fish waste bio-fertilizer on shoot potassium (K) and root calcium (Ca) of *Stevia rebaudiana* Bertoni. The same letters do not differ significantly ($P < 0.05$) based on Duncan 's test.

Treatment	Concentration (mM)	Shoot k (%)	Root Ca (%)
NaCl (mM)	0	4.58^{a}	0.323^{a}
	20	3.5^{b}	0.18^{b}
	40	2.62^{c}	0.14^{c}
	60	1.32^{d}	0.097^{d}
Fish waste biofertilizer (%)	0	2.71^{b}	0.138^{b}
	5	2.94^{ab}	0.166^{a}
	10	3.08^{a}	0.171^{a}
	15	3.29^{a}	0.174^{a}
